# *DYSF* gene variant spectrum in Arab populations across eight countries: A systematic review

**DOI:** 10.17305/bb.2026.13439

**Published:** 2026-02-12

**Authors:** Fatimazahra Smaili, Khawla Zerrouki, Fatima Ezzahra Aouni, Mariam Tajir

**Affiliations:** 1 Laboratory of Epidemiology, Clinical Research and Public Health, Faculty of Medicine and Pharmacy, Mohammed First University, Oujda, Morocco; 2 Laboratory of Medical Genetics, Central Laboratory, Mohammed VI University Hospital, Oujda, Morocco

**Keywords:** Dysferlinopathy, *DYSF* mutations, Arab populations

## Abstract

Dysferlinopathies are a subset of autosomal recessive muscular dystrophies resulting from pathogenic variants in the dysferlin (*DYSF*) gene. The prevalence of dysferlinopathies remains inadequately defined. This review aims to elucidate the mutational spectrum of *DYSF* in Arab populations. A systematic search was conducted in PubMed, ScienceDirect, Scopus, and Web of Science up to September 15, 2025. We identified 48 unique *DYSF* variants documented in the literature across eight Arab countries, resulting in 49 country-entries due to one variant being reported in two countries. The distribution of reported variants was as follows: Saudi Arabia 32.7% (16/49), Algeria 20.4% (10/49), Egypt 20.4% (10/49), Tunisia 10.2% (5/49), Morocco 6.1% (3/49), Libya 4.1% (2/49), Lebanon 4.1% (2/49), and Oman 2.0% (1/49). Clinical presentations were categorized based on phenotype assignments across variants, totaling 52 assignments as some variants were associated with multiple phenotypes: limb-girdle muscular dystrophy, recessive type 2 (LGMDR2) 50% (26/52), proximodistal 15% (8/52), Miyoshi myopathy 8% (4/52), distal myopathy with anterior tibial onset (DMAT) 4% (2/52), and asymptomatic hyperCKemia 4% (2/52). In terms of molecular consequences (denominator = 48 unique variants), frameshift variants constituted 36% (17/48), missense variants 29% (14/48), nonsense variants 15% (7/48), splice donor variants 6% (3/48), splice acceptor variants 4% (2/48), intronic variants 2% (1/48), and synonymous variants 2% (1/48). Documenting these variants across populations facilitates diagnosis and informs future public health strategies. Notably, no cohort study based in Morocco has focused on the genetics of dysferlinopathy; existing Moroccan data are limited to isolated case reports.

## Introduction

Dystrophy-associated fer-1-like protein, commonly referred to as human dysferlin, is a large transmembrane protein with a molecular weight of 238 kDa that belongs to the Ferlin protein family [1–10]. Dysferlin plays a crucial role in calcium signaling, sarcomere stability, membrane fusion, and repair [11–14].

The amino acid sequence of dysferlin is similar to that of the fer-1 protein found in the nematode Caenorhabditis elegans [3, 15–19].

The gene symbol for dysferlin is DYSF, as indicated in the Online Mendelian Inheritance in Man (OMIM) database under entry 603009. This extensive gene is located on chromosome 2 (p13.3) and spans over 150 kb of genomic DNA, with coding regions constituting only 1.67% distributed across 55 exons [1, 20, 21].

Dysferlinopathies represent a group of rare autosomal recessive muscular dystrophies caused by pathogenic variants in the *DYSF* gene, characterized by a heterogeneous spectrum of muscle disorders. Currently, the clinical presentations of Miyoshi Myopathy and limb-girdle muscular dystrophy (LGMD) type R2 are considered manifestations of the same condition, as the same mutation can lead to both clinical presentations. Additional minor symptoms associated with dysferlinopathy include asymptomatic hyperCKemia, distal myopathy with anterior tibial onset (DMAT), proximodistal (PD) myopathy, and pseudometabolic myopathy [22–24].

Since the identification of the first mutation in the *DYSF* gene in 1998 [25], a total of 909 pathogenic or likely pathogenic variants have been reported in the ClinVar database (http://www.ncbi.nlm.nih.gov/clinvar/, last accessed on January 7, 2026), with over 700 variants documented in the Leiden Open Variation Database (LOVD: https://databases.lovd.nl/shared/genes/DYSF). Establishing a genotype-phenotype correlation is challenging due to the diverse clinical phenotypes observed in dysferlinopathy patients [26–28].

A regional approach to identifying genetic mutations in the *DYSF* gene within the Arab population is essential. Disparities in diagnosis in these populations, particularly due to unequal access to molecular diagnostic tools, may result in significant underestimation of the true prevalence of dysferlinopathies. Additionally, the high frequency of consanguinity in certain countries contributes to the presence of founder mutations. Identifying these recurrent mutations enhances the accuracy of genetic diagnoses, facilitates targeted screening strategies, and optimizes genetic counseling and family care [29–33].

The objective of this review is to highlight the mutational spectrum of the *DYSF* gene in the Arab world, particularly in Morocco, based on literature data and our hospital database of patients affected by dysferlinopathy.

## Materials and methods

### Search strategy

The literature search was conducted using the PubMed, ScienceDirect, Scopus, and Web of Science databases. Our research utilized the following keywords: “Dysferlinopathy” OR “Dysferlinopathie” AND “*DYSF* gene mutation in Morocco” OR “*DYSF* gene mutation in Tunisia” OR “*DYSF* gene mutation in Algeria” OR “*DYSF* gene mutation in Saudi Arabia” OR “*DYSF* gene mutation in Lebanon” OR “*DYSF* gene mutation in Libya” OR “*DYSF* gene mutation in Egypt” OR “*DYSF* gene mutation in Oman” OR “*DYSF* gene mutation in Mauritania” OR “*DYSF* gene mutation in Sudan” OR “*DYSF* gene mutation in Djibouti” OR “*DYSF* gene mutation in Somalia” OR “*DYSF* gene mutation in Yemen” OR “*DYSF* gene mutation in United Arab Emirates” OR “*DYSF* gene mutation in Qatar” OR “*DYSF* gene mutation in Bahrain” OR “*DYSF* gene mutation in Kuwait” OR “*DYSF* gene mutation in Iraq” OR “*DYSF* gene mutation in Syria” OR “*DYSF* gene mutation in Jordan” OR “*DYSF* gene mutation in Palestine” OR “*DYSF* gene mutation in Comoros” AND “Dysferlin” AND “Ferlin protein” OR “Ferlin” AND “Arab populations” AND “*DYSF*” OR “*DYSF* gene mutation c.5594delG” OR “c.5594delG”.

**Figure 1.**
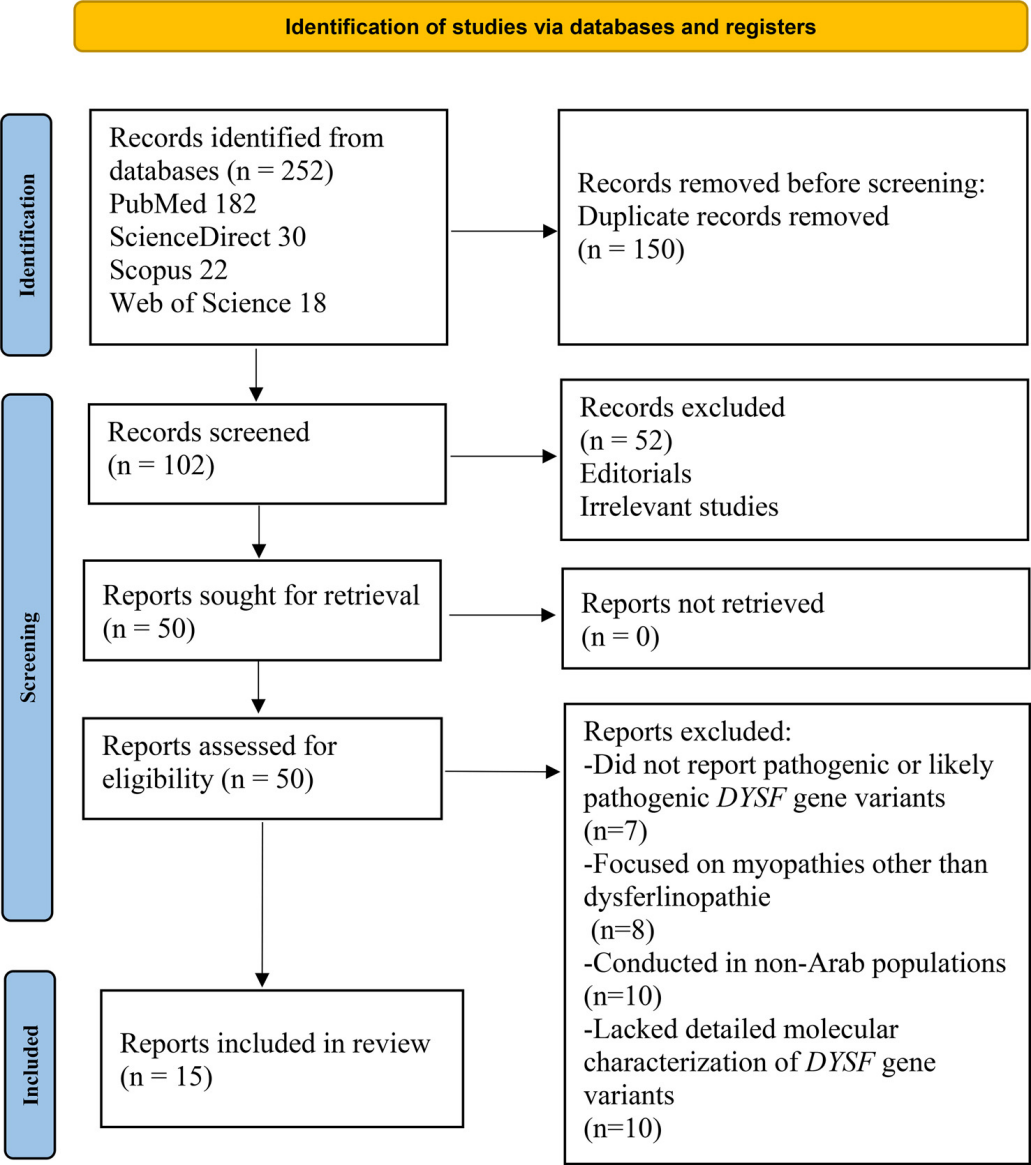
PRISMA diagram illustrating the process of research selection and study inclusion [34].

A total of 252 potentially relevant records in English and French were identified through database searches and citation screening. After removing 150 duplicates, 102 records remained for title and abstract screening, leading to the exclusion of 52 articles that did not align with the review objectives. The full texts of the remaining 50 articles were independently assessed by three reviewers, resulting in the exclusion of 35 studies. Ultimately, 15 studies met the inclusion criteria and were incorporated into the review (Figure 1).

The literature search was not limited to full-length peer-reviewed articles; it also included published abstracts. The search encompassed literature from 1980 through September 15, 2025.

### Eligibility criteria

Studies included in this review were those reporting clinical and genetic data from patients diagnosed with dysferlinopathy in Arab populations, with documented variants in the *DYSF* gene. Publications describing the molecular structure and functional characteristics of the dysferlin protein, as well as cohort studies involving patients affected by dysferlinopathy, were also included. Variant information was gathered from relevant curated genetic databases, including LOVD and ClinVar.

Exclusion criteria encompassed publications that did not report pathogenic or likely pathogenic variants in the *DYSF* gene in individuals from Arab populations, studies focusing on myopathies unrelated to dysferlinopathy, research conducted in non-Arab populations, and publications lacking detailed molecular characterization of *DYSF* gene variants.

Two reviewers independently performed study selection based on predefined inclusion and exclusion criteria. Disagreements were resolved by consensus or, when necessary, through consultation with a third reviewer. The details of the data extraction are presented in Table S1.

### Risk of bias assessment

This systematic review adhered to a predefined protocol in accordance with the Preferred Reporting Items for Systematic Reviews and Meta-Analyses (PRISMA) 2020 statement [34]. To evaluate the potential risk of bias in the included studies, reviewers assessed methodological quality using the Joanna Briggs Institute (JBI) Critical Appraisal Checklist for Case Reports and Case Series [35–37]. The JBI checklist comprises 10 items for case series and 8 items for case reports, with results displayed in Tables S2 and S3.

### Procedure for counting identified mutations

The analysis of mutations was based on the various variants reported by country.

We identified a total of 48 distinct mutations across 8 countries. Compound heterozygous mutations were considered separately, eliminating duplicates. The analysis and interpretation of data to determine molecular consequences, clinical classification, and information from the LOVD and ClinVar databases followed the same principles.

The classification of the reported variants was based on published data, supplemented by direct consultation of the LOVD and ClinVar databases, which were last accessed on January 7, 2026.

This study aimed to conduct a targeted and detailed investigation of the various mutations of the *DYSF* gene in the Arab world as reported in the literature. During our research, we formulated several questions that guided our exploration:

- What are the different mutations of the *DYSF* gene described in scientific publications that have been identified in Arab patients?
- Are there similarities among Arab countries regarding the published mutations of the *DYSF* gene?

In Morocco:

- What mutations have been identified in the *DYSF* gene?
- In how many patients has a mutation in the *DYSF* gene been identified?
- How many references to the *DYSF* gene have been cited in Morocco?
- Is there a founder effect associated with a mutation in the *DYSF* gene in the Moroccan population?

## Results

According to our literature review, forty-eight mutations in the *DYSF* gene have been reported in Arab populations. All analyses pertaining to molecular consequences and results from LOVD/ClinVar were performed using 48 as the denominator (unique variants), while analyses related to clinical classification, phenotype, and geographical origin were based on 49 entries (as one mutation occurs in two countries). The denominator for clinical presentations is 52, as some mutations are associated with multiple phenotypes. Fifteen publications [19, 29–31, 38–48] have described mutations of the *DYSF* gene in Saudi Arabia, Egypt, Algeria, Tunisia, Morocco, Libya, Lebanon, and Oman. To date, there are no reports of mutations in the *DYSF* gene for other countries, including Mauritania, Sudan, Djibouti, Somalia, Yemen, United Arab Emirates, Qatar, Bahrain, Kuwait, Iraq, Syria, Jordan, Palestine, and Comoros.

**Figure 2.**
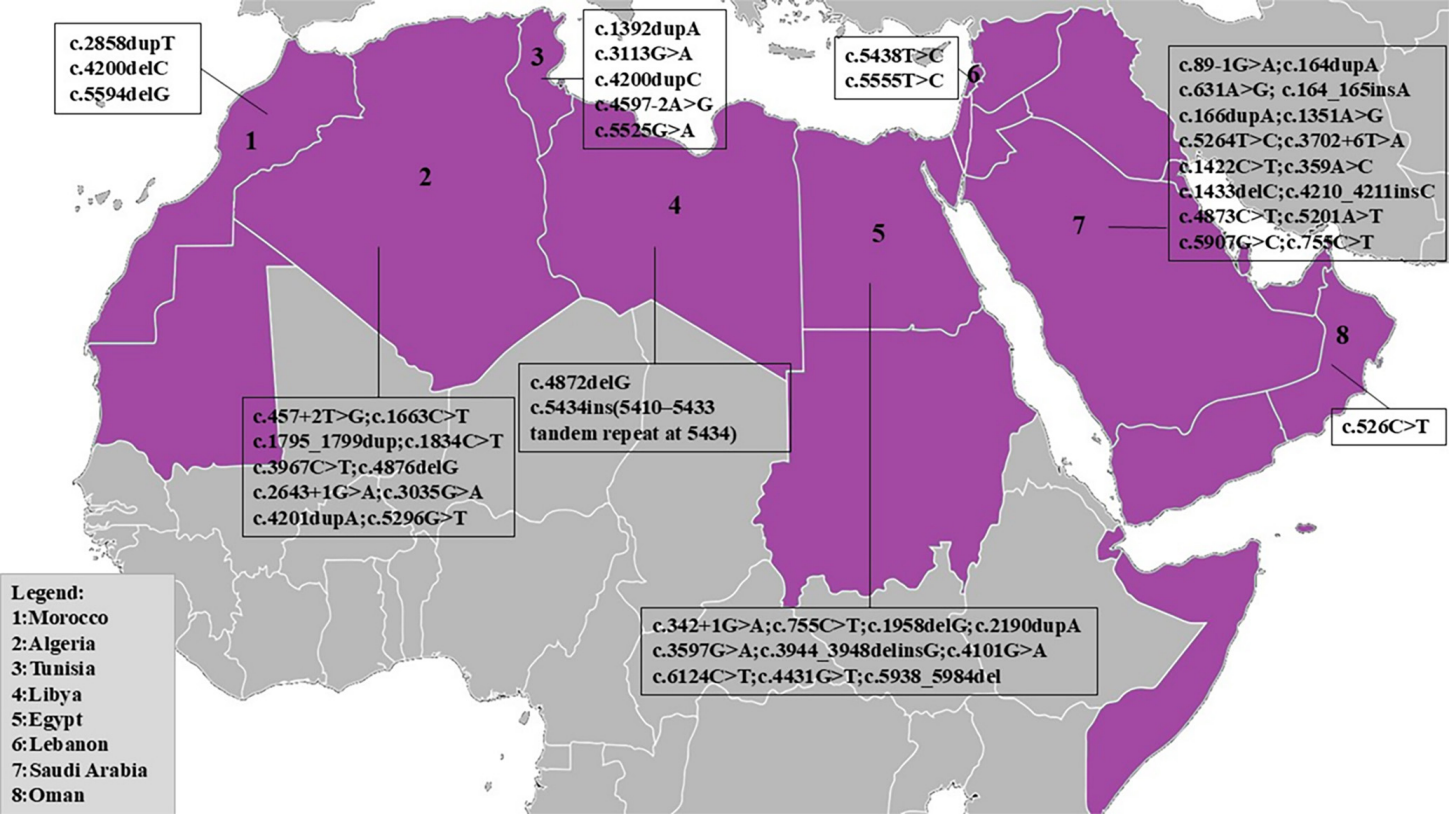
**Geographical distribution of reported *DYSF* variants in Arab countries.** Arab countries are shaded in purple; the eight countries with published *DYSF* variants included in this review are numbered as follows: 1 Morocco, 2 Algeria, 3 Tunisia, 4 Libya, 5 Egypt, 6 Lebanon, 7 Saudi Arabia, and 8 Oman. For each numbered country, the corresponding box lists the *DYSF* variants reported in the literature, presented using HGVS cDNA nomenclature (prefix “c.”). Overall, 48 unique variants were identified across these eight countries, yielding 49 country-entries because the c.755C>T variant was reported in both Saudi Arabia and Egypt; no published *DYSF* variants were identified for other Arab countries within the search timeframe (through 15 September 2025). Abbreviations: cDNA: Complementary DNA; *DYSF*: Dysferlin; HGVS: Human Genome Variation Society.

Sixteen mutations have been reported in Saudi Arabia, including 11 substitutions: c.89-1G>A; c.359A>C (p.Gln120Pro); c.631A>G (p.Lys211Glu); c.755C>T (p.Thr252Met); c.1351A>G (p.Met451Val); c.1422C>T; c.3702+6T>A; c.4873C>T (p.Arg1625*); c.5201A>T (p.Tyr1734Phe); c.5264T>C (p.Leu1755Pro); and c.5907G>C (p.Trp1969Cys). Additionally, there are two duplications: c.164dupA (p.Ile57Hisfs*8) and c.166dupA (p.L55fs); two insertions: c.164_165insA and c.4210_4211insC (p.Lys1404Thrfs*5); and one deletion: c.1433delC (Table 1, Table S4, and Figure 2).

**Table 1:** Summary of *DYSF* gene mutation data reported in arab populations from published studies

**Mutations**	**Protein change**	**Exon**	**Molecular consequence**	**Zygosity**	**Clinical classification**	**Phenotype**	**LOVD**	**ClinVar**	**Geographical origin**	**Number of patients**	**Reference**
c.89-1G>A	NR	2	Splice acceptor	Homozygous	Pathogenic	LGMDR2	√	√	Saudi Arabia	1 (1 family)	[31]
c.164dupA	p.Ile57Hisfs*8	3	Frameshift	Homozygous	Pathogenic	LGMDR2	√	√	Saudi Arabia	1 (1 family)	[42]
c.631A>G	p.Lys211Glu	6	Missense	Homozygous	Pathogenic	LGMDR2	NR	√	Saudi Arabia	1 (1 family)	[31]
c.164_165insA	NR	3	Frameshift	Homozygous	Pathogenic	LGMDR2	NR	NR	Saudi Arabia	28 (19 families)	[31]
c.166dupA	p.L55fs	3	Frameshift	Homozygous	Pathogenic	LGMDR2	NR	NR	Saudi Arabia	1 (1 family)	[41]
c.1351A>G	p.Met451Val	14	Missense	Compound heterozygous	VUS	LGMDR2	√	√	Saudi Arabia	1 (1 family)	[31]
c.5264T>C	p.Leu1755Pro	38	Missense		NR		NR	NR	Saudi Arabia		
c.3702+6T>A	NR	2	Intronic		NR		NR	√	Saudi Arabia		
c.1422C>T	NR	16	Silent	Compound heterozygous	Likely pathogenic	LGMDR2	NR	NR	Saudi Arabia	1 (1 family)	[31]
c.359A>C	p.Gln120Pro	16	Missense		Likely pathogenic		NR	√			
c.1433delC	NR	16	Frameshift	Homozygous	Pathogenic	LGMDR2	NR	NR	Saudi Arabia	3 (2 families)	[31]
c.4210_4211insC	p.Lys1404Thrfs*5	39	Frameshift	Homozygous	Pathogenic	LGMDR2	NR	NR	Saudi Arabia	1 (1 family)	[31]
c.4873C>T	p.Arg1625*	NR	Nonsense	Homozygous	Pathogenic	LGMDR2	NR	√	Saudi Arabia	NR	[43]
c.5201A>T	p.Tyr1734Phe	46	Missense	Homozygous	NR	LGMDR2	NR	√	Saudi Arabia	1 (1 family)	[31]
c.5907G>C	p.Trp1969Cys	52	Missense	Homozygous	VUS	LGMDR2	√	√	Saudi Arabia	1 (1 family)	[31]
c.755C>T	p.Thr252Met	7	Missense	Homozygous	VUS	LGMDR2	√	√	Saudi Arabia	1 (1 family)	[31]
c.342+1G>A	NR	Intron 4	Splice donor	Homozygous	Pathogenic	NR	√	√	Egypt		[45]
c.755C>T	p.Thr252Met	7	Missense	Homozygous	Pathogenic	NR	√	√	Egypt		[45]
c.1958delG	p.Gly653Valfs*3	21	Frameshift	Homozygous	Pathogenic	NR	√	NR	Egypt		[45]
c.2190dupA	p.Pro731Thrfs*23	NR	NR	Compound heterozygous	NR	NR	NR	NR	Egypt	21 patients	[45]
c.3597G>A	p.Trp1199*	33	Nonsense		Pathogenic	NR	√	√	Egypt		
c.3944_3948delinsG	p.Glu1315Glyfs*29	NR	NR	NR	NR	NR	NR	NR	Egypt		[45]
c.4101G>A	NR	NR	NR	Compound heterozygous	NR	NR	NR	NR	Egypt		[45]
c.6124C>T	p.Arg2042Cys	54	Missense		Pathogenic	NR	√	NR	Egypt		
c.4431G>T	p.Trp1477Cys	NR	Missense	Homozygous	Pathogenic	NR	√	NR	Egypt		[45, 46]
c.5983_5984del	p.Ser1995*	NR	Frameshift	Homozygous	NR	NR	NR	NR	Egypt		[45]
c.457+2T>G	p.Ala115_Asp153	Intron 5	Splice donor	Homozygous	Pathogenic	LGMDR2	√	√	Algeria	1 (1 family)	[38]
c.1663C>T	p.Arg555Trp	19	Missense	Homozygous	Pathogenic	MM	√	√	Algeria	1 (1 family)	[38]
c.1795_1799dup	p.Ala601Thrfs*28	20	Frameshift	Homozygous	Pathogenic	MM	√	NR	Algeria	2 (1 family)	[38]
c.1834C>T	p.Gln612*	20	Nonsense	Compound heterozygous	Pathogenic	LGMDR2	√	√	Algeria		[38]
c.3967C>T	p.Gln1323*	37	Nonsense		Pathogenic		√	NR	Algeria	2 (1 family)	
c.1834C>T	p.Gln612*	20	Nonsense		Pathogenic	PD phenotype	√	√	Algeria		
c.4876delG	p.Val1626Tyrfs*8	44	Frameshift	Compound heterozygous	Pathogenic		√	NR	Algeria		
c.2643+1G>A	p.Tyr838_Thr881del	Intron 25	Splice donor	Homozygous	Pathogenic	LGMDR2	√	√	Algeria	2 (1 family)	[40]
c.3035G>A	p.Trp1012*	29	Nonsense	Homozygous	Pathogenic	LGMDR2	√	√	Algeria	2 (1 family)	[38]
c.4201dupA	p.Ile1401Asnfs*8	39	Frameshift	Homozygous	Pathogenic	PD phenotype	√	NR	Algeria	1 (1 family)	[38]
c.5296G>T	p.Glu1766*	47	Nonsense	Homozygous	Pathogenic	PD phenotype	√	√	Algeria	1 (1 family)	[38]
c.1392dupA	p.Asp465Argfs*9	15	Frameshift	Homozygous	Pathogenic	-DMAT -PD phenotype	√	√	Tunisia	4 (2 families:3/1)	[29, 38]
c.3113G>A	p. Arg1038Gln	29	Missense	Homozygous	Likely pathogenic	-HyperCKemia -LGMDR2 -PD phenotype	√	√	Tunisia	9 (3 families: 6/2/1)	[29]
c.4200dupC	p.Ile1401Hisfs*8	39	Frameshift	Homozygous	Pathogenic	-LGMDR2 -MM -HyperCKemia	√	√	Tunisia	4 (2 families: 1/3)	[29]
c.4597-2A>G	NR	43	Splice acceptor	Homozygous	Pathogenic	-DMAT -PD phenotype -LGMDR2	NR	NR	Tunisia	3 (1 family)	[29, 39]
c.5525G>A	p.Gly1842Asp	49	Missense	Homozygous	Pathogenic	PD phenotype	√	NR	Tunisia	1 (1 family)	[29]
c.2858dupT	p.Phe954Valfs*2	27	Frameshift	Homozygous	Pathogenic	LGMDR2	√	√	Morocco	1 (1 family)	[38, 47]
c.4200delC	p.Ile1401Serfs*47	39	Frameshift	Homozygous	Pathogenic	LGMDR2	√	√	Morocco	1 (1 family)	[38]
c.5594delG	p.Gly1865Alafs*101	50	Frameshift	Homozygous	Pathogenic	PD phenotype	√	√	Morocco	1 (1 family)	[38, 47]
c.4872delG	p.Glu1624Aspfs*10	44	Frameshift	Homozygous	Pathogenic	LGMDR2	√	√	Libya	29 (12 families)	[30]
c.5434ins (5410–5433 tandem repeat at 5434)	NR	46	Frameshift	Homozygous	Pathogenic	LGMDR2	NR	NR	Libya	NR	[19]
c.5438T>C	p.Leu1813Pro	49	Missense	Homozygous	Pathogenic	LGMDR2	√	NR	Lebanon	2 (2 families)	[44]
c.5555T>C	p.Lys1852Pro	50	Missense	Homozygous	Likely pathogenic	LGMDR2	NR	√	Lebanon	NR	[43]
c.526C>T	p.Gln176*	6	Nonsense	Homozygous	Pathogenic	MM	√	√	Oman	NR	[48]

Variant nomenclature was verified using several databases, including LOVD, ClinVar, VariantValidator (https://variantvalidator.org/), VarSome (https://varsome.com/), and Mutalyzer (https://mutalyzer.nl), all accessed on January 7, 2026. Abbreviations: LGMDR2: Limb-girdle muscular dystrophy, recessive type 2; MM: Miyoshi myopathy; DMAT: Distal myopathy with anterior tibial onset; PD: Proximodistal; VUS: Variant of uncertain significance; √: Found; NR: Not reported.

In Egypt, ten mutations have been reported, comprising six substitutions: c.342+1G>A; c.755C>T (p.Thr252Met); c.3597G>A (p.Trp1199*); c.4101G>A; c.4431G>T (p.Trp1477Cys); and c.6124C>T (p.Arg2042Cys). There are also two deletions: c.1958delG (p.Gly653Valfs*3) and c.5983_5984del (p.Ser1995*); one duplication: c.2190dupA (p.Pro731Thrfs*23); and one deletion-insertion mutation: c.3944_3948delinsG (p.Glu1315Glyfs*29) (Table 1, Table S4, and Figure 2).

Algeria has identified ten different mutations in the *DYSF* gene, including seven substitutions: c.457+2T>G (p.Ala115_Asp153); c.1663C>T (p.Arg555Trp); c.1834C>T (p.Gln612*); c.2643+1G>A (p.Tyr838_Thr881del); c.3035G>A (p.Trp1012*); c.3967C>T (p.Gln1323*); and c.5296G>T (p.Glu1766*). Additionally, there are two duplications: c.1795_1799dup (p.Ala601Thrfs*28) and c.4201dupA (p.Ile1401Asnfs*8); and one deletion: c.4876delG (p.Val1626Tyrfs*8) (Table 1, Table S4, and Figure 2).

In Tunisia, five different mutations in the *DYSF* gene have been reported, consisting of three substitutions: c.3113G>A (p.Arg1038Gln); c.4597-2A>G; and c.5525G>A (p.Gly1842Asp) along with two duplications: c.1392dupA (p.Asp465Argfs*9) and c.4200dupC (p.Ile1401Hisfs*8) (Table 1, Table S4, and Figure 2).

Morocco has identified three mutations: two deletions, c.5594delG (p.Gly1865Alafs*101) and c.4200delC (p.Ile1401Serfs*47), and one duplication, c.2858dupT (p.Phe954Valfs*2) (Table 1, Table S4, and Figure 2).

In the Lebanese population, two substitutions have been reported: c.5438T>C (p.Leu1813Pro) and c.5555T>C (p.Lys1852Pro) (Table 1, Table S4, and Figure 2).

Libya has reported two mutations: one deletion, c.4872delG (p.Glu1624Aspfs*10), and one insertion, c.5434ins (5410_5433 tandem repeat at 5434) (Table 1, Table S4, and Figure 2).

In Oman, one nonsense mutation has been described: c.526C>T (p.Gln176*) (Table 1, Table S4, and Figure 2).

**Figure 3.**
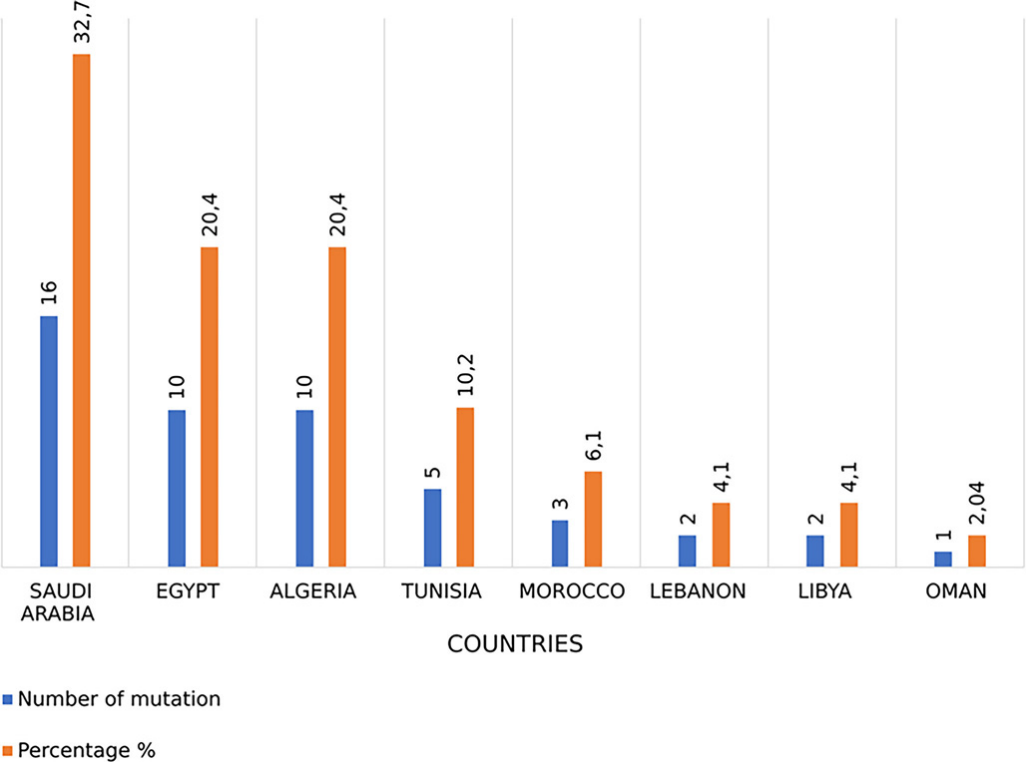
**Distribution of reported *DYSF* variants across Arab countries.** Blue bars indicate the number of reported *DYSF* variant country-entries per country, and orange bars indicate the corresponding percentage of all country-entries identified in this review (total = 49). Percentages were calculated using country-entries rather than unique variants because one variant (c.755C>T) was reported in both Saudi Arabia and Egypt. Saudi Arabia contributed the highest proportion (16/49, 32.7%), followed by Egypt and Algeria (10/49 each, 20.4%), Tunisia (5/49, 10.2%), Morocco (3/49, 6.1%), Lebanon and Libya (2/49 each, 4.1%), and Oman (1/49, 2.0%). Abbreviation: *DYSF*: Dysferlin.

This review indicates that Saudi Arabia has the highest percentage of reported *DYSF* mutations (32.7%), followed by Algeria (20.4%), Egypt (20.4%), Tunisia (10.2%), Morocco (6.1%), Lebanon (4.1%), Libya (4.1%), and Oman (2%) (Figure 3).

Only one variant overlap was observed among the studied countries, specifically c.755C>T, identified in both Saudi Arabia and Egypt. All other variants were unique to their respective countries.

**Figure 4.**
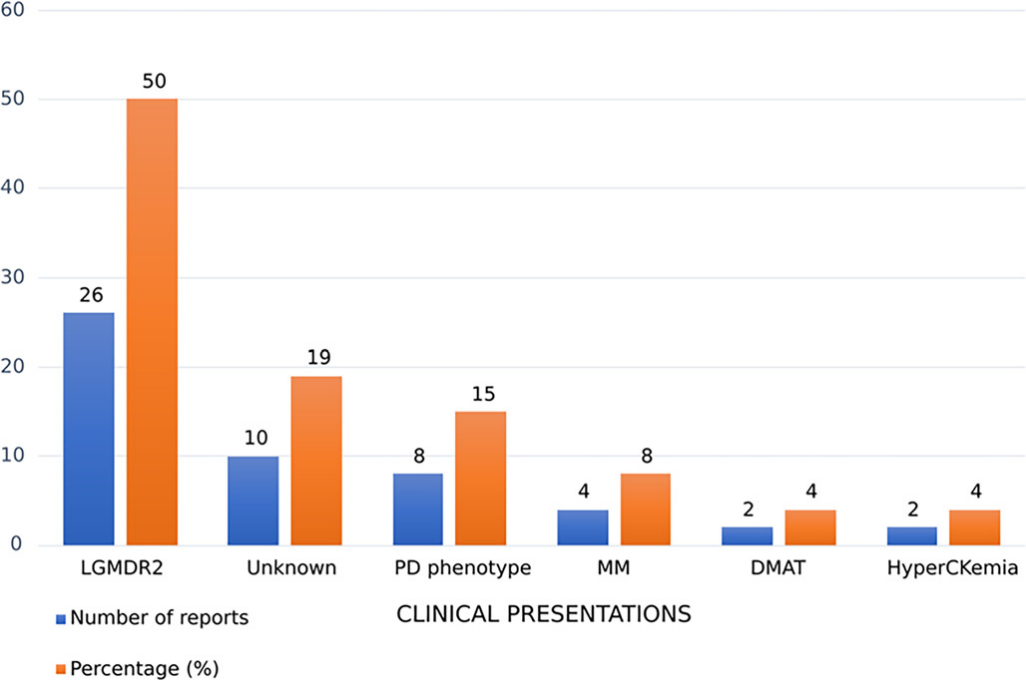
**Clinical presentation distribution across reported *DYSF* variant phenotype assignments in Arab populations.** Blue bars indicate the number of phenotype assignments (“reports”) and orange bars indicate the corresponding percentage of all phenotype assignments identified in this review (total = 52); percentages sum to 100% because some variants were associated with more than one clinical presentation. LGMDR2 was the most frequent presentation (26/52, 50%), followed by PD phenotype (8/52, 15%), MM (4/52, 8%), DMAT (2/52, 4%), and hyperCKemia (2/52, 4%). In 10/52 assignments (19%), the clinical presentation was not specified in the source report (Unknown). Abbreviations: *DYSF*: Dysferlin; DMAT: Distal myopathy with anterior tibial onset; LGMDR2: Limb-girdle muscular dystrophy, recessive type 2; MM: Miyoshi myopathy; PD: Proximodistal; hyperCKemia: Hypercreatine kinaseemia.

The most prevalent clinical presentation of dysferlinopathy in our study is LGMD type 2 (LGMDR2) (50%), followed by the PD phenotype (15%), miyoshi myopathy (MM) (8%), DMAT (4%), and asymptomatic hyperCKemia (4%). The specific type of dysferlinopathy remained unknown for 19% of the reported mutations (Figure 4).

**Figure 5.**
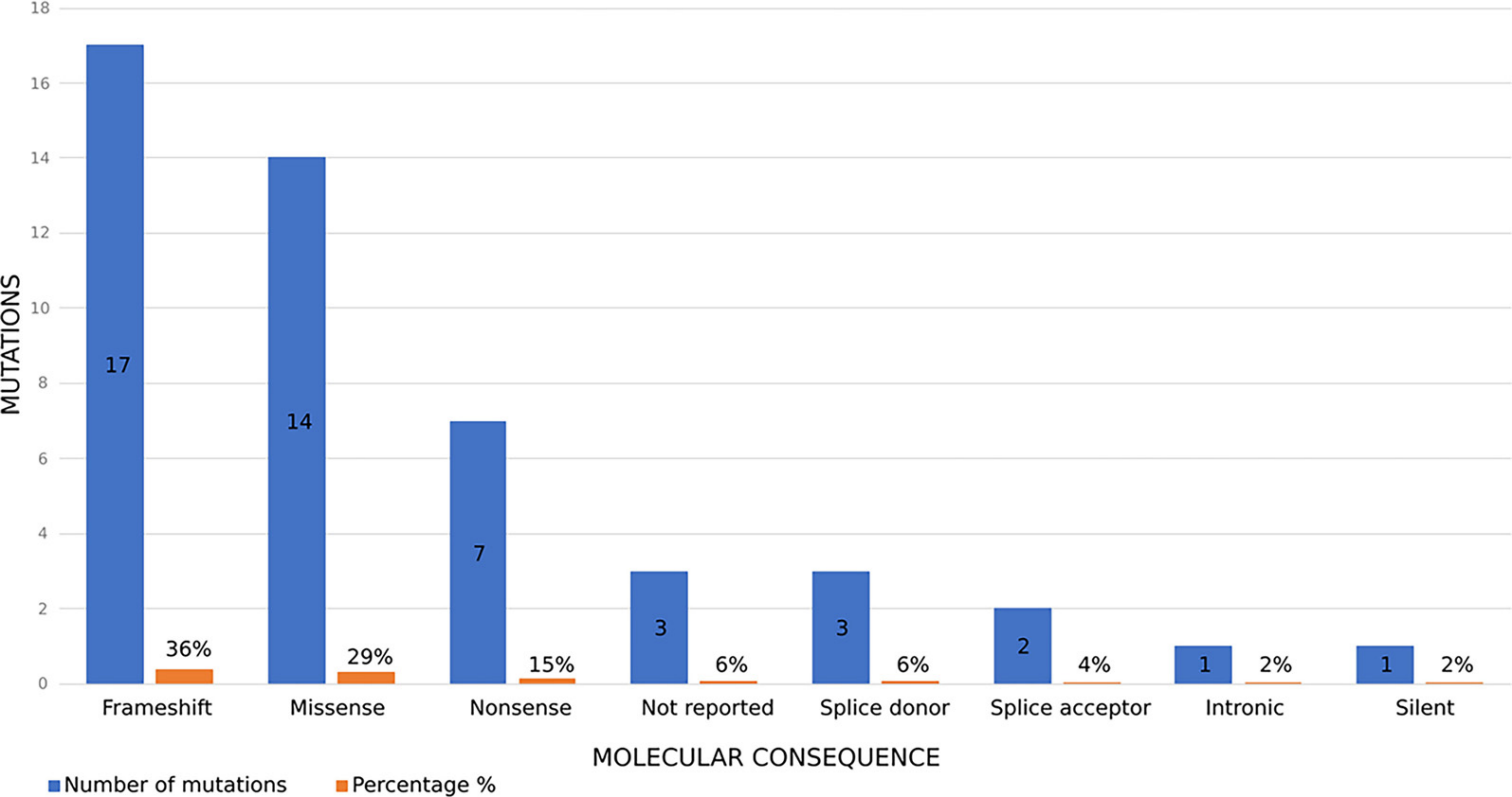
**Distribution of reported *DYSF* variants by predicted molecular consequence.** Blue bars indicate the number of unique variants in each consequence category and orange bars indicate the corresponding percentage of all unique variants identified in this review (total = 48). Frameshift variants were most frequent (17/48, 36%), followed by missense (14/48, 29%) and nonsense (7/48, 15%) variants. Splice-site variants accounted for 6% (splice donor, 3/48) and 4% (splice acceptor, 2/48). Intronic and synonymous (“silent”) variants each accounted for 2% (1/48). For 3/48 variants (6%), the molecular consequence was not reported in the source publication (Not reported). Abbreviation: *DYSF*: Dysferlin.

The distribution of disease-causing mutations identified in the literature among patients of Arab origin is as follows: 36% frameshift, 29% missense, 15% nonsense, 6% splice donor, and 4% splice acceptor mutations. Intronic and silent mutations account for 2% each, while 6% of mutations were unreported (Figure 5).

**Figure 6.**
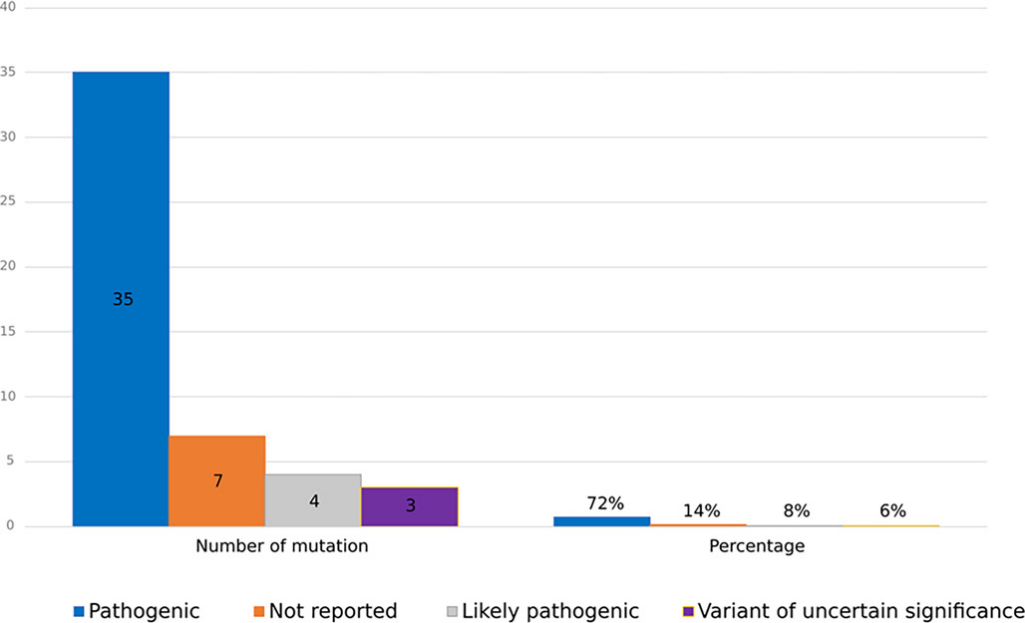
**Distribution of reported *DYSF* variants by clinical classification.** Bars show the number (left) and percentage (right) of unique *DYSF* variants assigned to each clinical classification category (total = 48). Most variants were classified as pathogenic (35/48, 72%), followed by likely pathogenic (4/48, 8%) and variants of uncertain significance (3/48, 6%). For 7/48 variants (14%), a clinical classification was not reported in the source publication and no curated classification was identified during database cross-checking (Not reported). Classifications were extracted primarily from the original reports and, when necessary, supplemented by evidence from LOVD and ClinVar. Abbreviations: *DYSF*: Dysferlin; LOVD: Leiden Open Variation Database; VUS: Variant of uncertain significance.

The clinical classification of the various mutations shows a predominance of pathogenic mutations (72%), followed by likely pathogenic mutations (8%) and variants of uncertain significance (6%). Fourteen percent of the mutations lacked any reported clinical classification (Figure 6). The initial classification source was based on articles reporting the mutations, supplemented by research in the LOVD and ClinVar.

**Figure 7.**
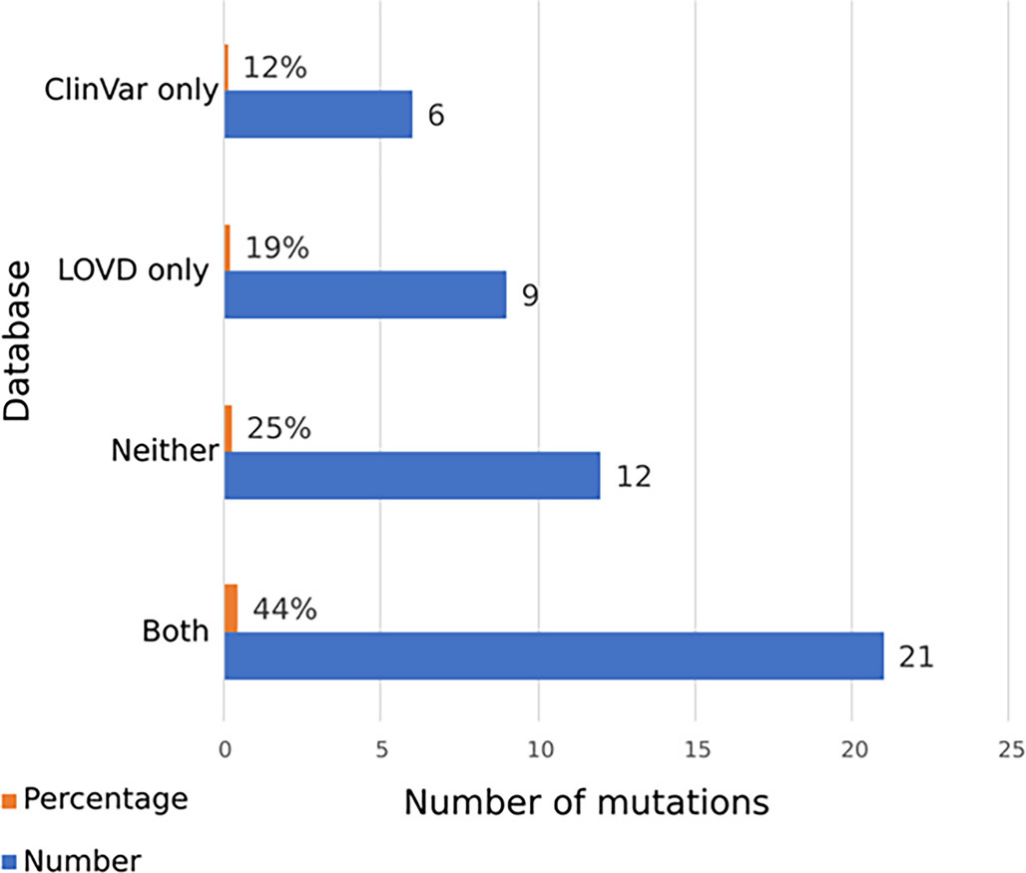
**Presence of reported *DYSF* variants in curated variant databases (LOVD and ClinVar).** Horizontal bars show the number (blue) and percentage (orange) of unique *DYSF* variants identified in this review (total = 48) that were present in both databases (Both), in only one database (LOVD only or ClinVar only), or in neither database (Neither). Twenty-one variants (21/48, 44%) were found in both LOVD and ClinVar, 9/48 (19%) were found in LOVD only, 6/48 (12%) were found in ClinVar only, and 12/48 (25%) were not identified in either database at the time of querying. Database searches were performed on 7 January 2026. Abbreviations: *DYSF*: Dysferlin; LOVD: Leiden Open Variation Database.

The distribution of variants from the 48 analyzed mutations across the two databases is as follows: 21 (44%) were identified in both LOVD and ClinVar, 12 (25%) variants were not found in either database, 9 (19%) variants were found in LOVD only, and 6 (12%) in ClinVar only. The query dates used for these counts were January 7, 2026 (Figure 7).

### Study quality evaluation

As detailed in Table S2 and Table S3, the methodological quality of the included studies was assessed using the JBI Critical Appraisal Checklist. Although some concerns were noted based on the JBI appraisal, all articles were ultimately retained. Overall, the methodological quality of the included case series was moderate. Most studies clearly defined inclusion criteria and employed reliable diagnostic methods. However, the consecutive and complete inclusion of participants was often unclear, and follow-up information was inconsistently reported. These limitations are characteristic of descriptive case series, particularly in the context of rare diseases. The four included case reports demonstrated adequate methodological quality, featuring clear patient descriptions, reliable diagnostic methods, and well-documented clinical presentations. Some limitations were noted, particularly regarding follow-up information and the reporting of additional clinical details, which were not always applicable or fully addressed. Nevertheless, the included studies provide valuable clinical and genetic information that is essential for qualitative synthesis.

## Discussion

Dysferlinopathy constitutes a heterogeneous group of clinical disorders primarily characterized by two clinical presentations: LGMDR2, which manifests as proximal lower limb muscle weakness, and myopathy, characterized by distal muscle weakness in the lower limbs [49]. Although these two entities are now unified under the classification of dysferlinopathies [24], the literature often discusses them separately. The prevalence of dysferlinopathies is estimated to range from 1 in 14,000 to 1 in 2 million, making them one of the most common forms of LGMD [50].

A study conducted by Rochdi et al. identified the most frequently altered genes associated with myopathies in the Middle East and North Africa (MENA) population, specifically the *DYSF*, *SGCG*, and *LAMA2* genes [51]. In the studies included in this review, 48 unique *DYSF* variants were reported across Arab populations, with the LGMDR2 phenotype being the most frequently assigned (26 out of 52 cases). In Tunisia, Laroussi et al. reported that among 20 patients, the most common phenotypes were LGMDR2 and PD LGMD, each accounting for 35% of cases [52]. In contrast, a study in Saudi Arabia involving 53 subjects from 33 families found the LGMDR2 phenotype in 30.19%, the MM phenotype in 50.94%, and the PD phenotype in 15.09% [31].

The research by Alharbi et al. in Saudi Arabia identified 13 different mutations in patients from 29 unrelated families, with the c.164_165insA mutation being the most prevalent, affecting 28 patients from 19 unrelated families. The c.1433delC mutation was the second most frequent, observed in three patients from two unrelated families [31]. In Tunisia, five distinct mutations were reported among 21 patients from unrelated families, with the c.3113G>A (p.Arg1038Gln) mutation identified in nine patients, followed by c.4597-2A>G in three patients [29, 39]. In the Libyan Jewish community, 29 patients were found to carry the c.4872delG (p.Glu1624Aspfs*10) founder mutation [30].

Nguyen et al. reported three mutations in the *DYSF* gene among Moroccan patients: c.2858dupT (p.Phe954Valfs*2), c.5594delG (p.Gly1865Alafs*101), and c.4200delC (p.Ile1401Serfs*47) [38, 47]. In our Medical Genetics Laboratory at the Mohammed VI University Hospital Center of Oujda, the c.5594delG mutation was confirmed in a patient with dysferlinopathy, whose brother and two cousins also exhibited similar clinical features. This mutation has been identified in the two cousins (data not published; the Biomedical Research Ethics Committee of Oujda, Morocco (CERBO), approved the ethical protocol for this study). At the complementary DNA level, the c.5594delG mutation represents a deletion of guanine (G) at position 5594 in exon 50 of the *DYSF* gene. At the protein level, it results in the substitution of glycine for alanine at codon 1865, leading to a frameshift and premature termination of protein production after an additional 101 amino acids. This mutation has also been documented in Spanish and Belgian families. The recurrent frameshift mutation c.5594delG is not associated with a specific ethnic group or phenotype [47].

This review suggests that the c.5594delG mutation may represent a recurrent variant observed in multiple unrelated Moroccan patients. Further investigation through larger Moroccan cohorts or founder analyses in patients exhibiting clinical features suggestive of dysferlinopathy is warranted.

The Moroccan Genetic Disease Database (MGDD) does not currently include the c.4200delC (p.Ile1401Serfs*47) mutation, which should be added to facilitate ongoing research. The other two mutations have been included in the database [53].

The geographical distribution of mutations reported in the literature is influenced by demographic factors, including population size, consanguinity, access to molecular diagnostic techniques, and potential publication bias.

Demographically, Egypt has the largest population, estimated at around 116.5 million inhabitants in 2024, followed by Algeria, Morocco, and Saudi Arabia. This demographic distribution significantly influences the total number of reported cases (Table 2) [54, 55].

**Table 2:** Consanguinity and demographic profiles of Arab countries

**Countries**	**Overall consanguinty rate %**	**Population mid-2024 (millions)**
Egypt	20.9–80.4 [32]	116.5 [55]
Algeria	22.6–34 [32]	46.8 [55]
Saudi Arabia	42.1–66.7 [32]	34.0 [55]
Morocco	19.9–28 [32]	38.1 [55]
Tunisia	20.1–39.3 [32]	12.3 [55]
Libya	48.4 [32]	7.4 [55]

The high prevalence of certain autosomal recessive diseases, such as dystrophinopathies, within Arab populations can be partially attributed to the elevated rates of consanguineous marriages. This phenomenon is particularly documented in Saudi Arabia and Egypt, where consanguinity rates are among the highest globally (Table 2) [32, 33, 56].

While some countries have established national genomics projects and specialized centers (Saudi Arabia, Qatar, Egypt, and the United Arab Emirates), such initiatives are not yet widespread across all Arab nations [57].

In a cohort analysis of 134 patients with dysferlinopathy from various countries, Krahn et al. demonstrated the following distribution of mutation types: 28% frameshift mutations, 26% missense mutations, 26% nonsense mutations, 18% intronic mutations, and 1% in-frame deletions [58]. The study by Blandin et al. reported a total of 266 different types of mutations, categorized as follows: 27.8% frameshift mutations, 33.1% missense mutations, 18.0% nonsense mutations, and 3.8% in-frame exonic insertions or deletions [26]. In Nguyen et al.’s study of 34 patients with dysferlinopathy, the identified mutations included 17 missense mutations, 13 frameshift mutations, 13 nonsense mutations, 7 substitutions or small deletions at splice sites, 1 intronic one-base-pair substitution, and 3 in-frame insertions [47]. The mutations identified in our review include 36% frameshift mutations, 29% missense mutations, 15% nonsense mutations, 6% splice donor mutations, and 4% splice acceptor mutations, with intronic and silent mutations each representing 2%. Notably, the most prevalent types of disease-causing mutations are frameshift, missense, and nonsense mutations.

Several studies have indicated that there is no correlation between genotype and phenotype in dysferlinopathies. Even within families, identical mutations can lead to divergent phenotypes [50, 59, 60]. Moreover, in Arab populations, identified mutations are dispersed throughout the entire coding sequence of the *DYSF* gene, with no identifiable ‘hotspot’ mutations. Genotype alone is insufficient for predicting disease outcomes, as the same homozygous mutation can present with variable clinical manifestations. This suggests that factors beyond genetics, including environmental influences, may also modulate phenotypic expression [31, 58].

In the study by Rekik et al., a Tunisian family was identified with three members affected by dysferlinopathy, all sharing the same mutation, c.4597-2A>G, in the *DYSF* gene. This mutation resulted in the deletion of 28 bp from exon 43, leading to an intra-familial variety of dysferlinopathies, including the PD phenotype, LGMDR2, and DMAT [29, 39]. Similarly, Belhassen et al. reported that nine patients from three different families in Tunisia shared the homozygous missense mutation c.3113G>A, yet exhibited diverse phenotypes (LGMDR2, HyperCKemia, and PD). Additionally, the reading frameshift mutation c.4200dupC, found in two families, was associated with various phenotypes, including LGMDR2, MM, and HyperCKemia [29]. The c.5594delG mutation has also been linked to different phenotypes: PD in a Moroccan patient and Miyoshi myopathy in a Belgian patient [47].

In our dataset, the c.5594delG variant was identified in a single Moroccan family. While this variant has been documented in Moroccan patients in the literature, it has not yet been described in a Morocco-based cohort study; thus, the available evidence regarding Moroccan cases remains limited to isolated reports.

Compound heterozygous gene mutations refer to the presence of two or more heterogeneous recessive alleles at a specific locus, resulting in a genetic disease in the heterozygous state [31]. Our review identified six distinct compound heterozygous mutations. Two mutations, c.1834C>T/c.3967C>T and c.1834C>T/c.4876delG, were reported from Algeria, with one associated with the LGMDR2 phenotype and the other with the PD phenotype [38]. Two mutations from Saudi Arabia, c.1422C>T/c.359A>C and a triple heterozygous mutation c.1351A>G/c.5264T>C/c.3702+6T>A, both resulted in the LGMDR2 phenotype [31]. Additionally, two mutations, c.2190dupA/c.3597G>A and c.4101G>A/c.6124C>T, were reported in Egypt [45].

The LOVD, established in 1998 for dysferlin, serves as the only locus-specific database providing public reference for human dysferlin variants [26]. Our review revealed that 12 mutations were not documented in either the LOVD or ClinVar databases. These findings are significant as they indicate the limited global recognition of Arab mutations, underscoring the necessity to strengthen efforts in collecting and incorporating Arab mutations into these databases to enhance diagnosis and research. Consequently, creating comprehensive databases that encompass the various mutations found in Arab populations and ensuring their regular updates is essential.

### Limitations

This study has several limitations that should be considered when interpreting the results.

A comprehensive investigation is needed to establish a clearer understanding of the mutational profile of the *DYSF* gene in Morocco. This should involve collaboration with various genetic diagnostic centers throughout Morocco to gather comprehensive data on patients with dysferlinopathy.

Most of the included studies were conducted on small, heterogeneous samples.

There are potential biases related to selection and publication, as the patients included in studies may not represent the general population, and many studies tend to report positive mutation findings predominantly.

Finally, due to the limited number of studies and small sample sizes, a meta-analytic approach to data synthesis was not feasible, leading to a diminished quantitative synthesis.

## Conclusion

This review summarizes the mutations documented in eight Arab countries, reporting a total of 48 different variants of the *DYSF* gene, primarily consisting of frameshift and nonsense mutations. No mutational hotspots were identified.

To date, no Morocco-based cohort study on genetic dysferlinopathy has been published, though this study has reported an isolated Moroccan case. This highlights the urgent need for an Arab dysferlinopathy registry and inclusion in the LOVD/ClinVar databases.

Accurate characterization of the *DYSF* mutational spectrum is crucial for refining diagnostic strategies.

The observed differences in the number of patients with the *DYSF* gene mutations across various countries may be attributed to technological advancements in some regions compared to others, founder effects, patterns of consanguinity, registry activities, and practices surrounding referral and publication.

The absence of reported *DYSF* gene mutations in some countries likely reflects underdiagnosis, underreporting, and unequal access to sequencing, rather than a genuine absence of mutations or disease. Thus, this review offers a snapshot of published variants and should not be used to infer the prevalence or incidence of the disease.

## Supplemental data

Supplemental data are available at the following link: https://www.bjbms.org/ojs/index.php/bjbms/article/view/13439/4130.
